# Exploring Aneurysmal Bone Cysts of the Skull: Insights from a Review of the Literature and a Case Report

**DOI:** 10.3390/children12060715

**Published:** 2025-05-30

**Authors:** Flavio Panico, Leonardo Bradaschia, Pasquale Cardellicchio, Fabio Cofano, Enrico Lo Bue, Stefano Colonna, Alberto Morello, Andrea Bianconi, Diego Garbossa, Gianluca Piatelli, Marco Pavanello

**Affiliations:** 1 Neurosurgery Unit, Department of Neuroscience “Rita Levi Montalcini”, A.O.U. Città Della Salute e Della Scienza Torino, University of Turin, 10126 Turin, Italy; flavio.panico@unito.it (F.P.); fabio.cofano@unito.it (F.C.); enrico.lobue@unito.it (E.L.B.); stefano.colonna@unito.it (S.C.); alberto.morello@unito.it (A.M.); andrea.bianconi@unito.it (A.B.); diego.garbossa@unito.it (D.G.); 2 Physical Medicine and Rehabilitation Unit, IRCCS Istituto Giannina Gaslini, 16147 Genoa, Italy; pasquale.cardellicchio@gaslini.org; 3 Department of Neurosurgery, IRCCS Istituto Giannina Gaslini, 16147 Genoa, Italy; gianlucapiatelli@gaslini.org (G.P.); pavanellomarco.mp@gmail.com (M.P.)

**Keywords:** fibrous dysplasia, head trauma, skull bones, male hormones

## Abstract

**Background/Objectives**: Aneurysmal bone cysts (ABCs) are rare bone tumors that can occur in the skull, leading to extensive bone destruction and compression of surrounding tissues. Due to the rarity of these lesions, there are limited data available in the literature, which primarily consists of case reports. We aimed to collect and analyze the available data to summarize the current state of knowledge on this rare pathology, while also conducting a statistical analysis to identify potential risk factors and management strategies. **Methods**: A review was performed following the Preferred Reporting Items for Systematic Reviews and Meta-Analyses (PRISMA) guidelines, covering studies published from January 1950 to December 2023. A total of 60 articles and 74 case reports were included. **Results**: The mean age at diagnosis was 14.8 ± 12.5 years, with slightly higher male gender predominance. Regarding the different skull bones, a statistically significant higher growth trend of ABCs was found at the parietal bone in the male population (*p* = 0.025). At the occipital bone, a significant correlation was observed with the age of incidence for symptomatic lesions (*p* = 0.007) and development from fibrous dysplasia (*p* = 0.019). Secondary lesions showed a higher frequency of complications within the first months post-surgery (*p* = 0.041). **Conclusions**: No significant correlation was found between ABCs and fibrous dysplasia (FD) or head trauma. Male patients with FD showed a higher tendency to develop an aneurysmal cyst at the occipital bone at an older age and a higher tendency for growth in ABCs at the parietal bone. However, to date, no molecular or genetic correlation with male hormones has been reported in the literature. Surgery remains the only effective treatment, but complications should be carefully considered, particularly in patients with pre-existing pathological conditions.

## 1. Introduction

Aneurysmal bone cysts (ABCs) are rare, benign, vascular bone tumors most commonly diagnosed during the first two decades of life [1]. These complex lesions consist of blood-filled cystic spaces separated by fibrous stroma containing inflammatory cells, numerous capillaries, and multinucleated giant cells/osteoclasts [2]. The most typical location for an ABC is the metaphysis of long bones; therefore, lesions occurring in the calvaria are exceedingly rare, accounting for only 3–6% of all such vascular bone lesions [3].

Although benign, ABCs can lead to extensive bone destruction and compression of surrounding tissues due to their expansile nature, resulting in symptoms such as pain, swelling, deformity, pathological fractures, and neurological deficits. Most patients are treated with surgical curettage, but recurrence is common and may require additional surgical intervention.

To date, various risk factors in the development of ABCs have been investigated in the literature, with fibrous dysplasia and head trauma emerging as the most likely contributors to the pathogenesis of the disease.

We therefore aimed to collect and synthesize the available data to provide an up-to-date overview of this rare pathology, while also performing a statistical analysis to identify potential risk factors and optimal management strategies.

## 2. Materials and Methods

This review was conducted in accordance with the Preferred Reporting Items for Systematic Reviews and Meta-Analyses (PRISMA) guidelines [4]. Additionally, a single case report from our institution was included in the case history.

### 2.1. Objectives

The primary objective of this study is to investigate the existence of a correlation between the major risk factors currently implicated in the etiopathogenesis of ABC—namely, head trauma and fibrous dysplasia. Secondary objectives include exploring potential correlations between patient demographic data and the formation and progression of ABC lesions, particularly with respect to the bone of origin and their management.

### 2.2. Literature Search

All full-text, English-language manuscripts reporting relevant case reports of aneurysmal bone cysts (ABCs) were screened using the PubMed/MEDLINE, Embase, Cochrane Library, Scopus, and Web of Science databases, covering the period from February 1965 to December 2023. Search terms included key phrases and Boolean operators, such as “Aneurysmal bone“ OR “Aneurysmal cyst” AND “Fibrous dysplasia” OR “Skull bone” OR “Case report” OR “Trauma”. The Strengthening the Reporting of Observational studies in Epidemiology (STROBE) diagram illustrates the steps involved in screening and analyzing the articles selected for this review.

### 2.3. Study Selection

Articles were initially screened based on title, abstract, and full text to assess their eligibility, and any discrepancies were resolved by consensus. Studies were included if they met the following predefined inclusion criteria: they had to be case reports or case series written in English, refer specifically to aneurysmal bone cysts (ABCs) and not solely to fibrous dysplasia (FD), and report at least one relevant clinical outcome (it was not necessary for all outcomes of interest to be included).

Studies were excluded if they were not in English, not published in peer-reviewed journals, unrelated to aneurysmal bone cysts of the skull bones, or if they involved animal models, cadaveric specimens, or purely histological analyses. With regard to “skull bones”, only lesions affecting the neurocranium—namely, the frontal, parietal, temporal, ethmoid, sphenoid, and occipital bones—were included. Studies involving lesions of the maxilla, mandible, or zygomatic bones were excluded. Likewise, cases of ABCs extending into the orbital space or originating in the neurocranium but extending beyond the cranial vault were excluded.

Further exclusions included studies with unavailable full texts, insufficient demographic or treatment data, aggregated data, or anatomical locations outside the head. Duplicate reports of previously published cases were also excluded to avoid double counting.

### 2.4. Outcomes

A total of 586 manuscripts were identified during the initial phase of the literature search, of which 471 were excluded based on title and abstract screening. All remaining reports were successfully retrieved. Of the 115 full-text articles assessed for eligibility, the following were excluded: 13 written in languages other than English, 1 animal study, 1 radiological study, and 1 that did not include any case report or case series. An additional 39 articles were excluded due to the anatomical location not being relevant to the neurocranium.

**Figure 1:**
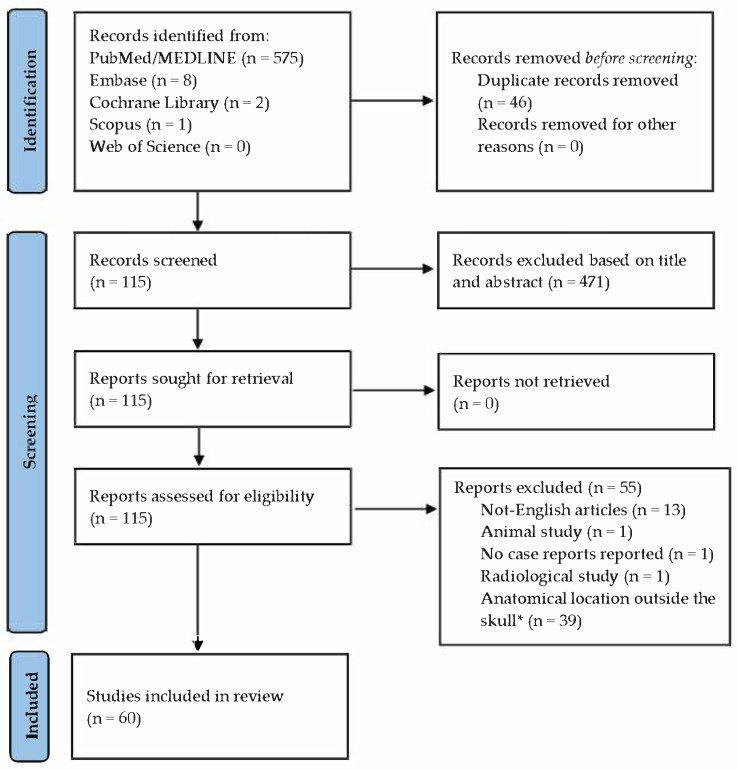
Articles’ selection following the PRISMA guidelines. Source: https://www.bmj.com/content/372/bmj.n71 (accessed on 15 April 2025). * The term “Skull” refers only to the neurocranium, and, therefore, to the bones forming it.

This process resulted in 60 articles meeting the inclusion criteria (Figure 1). The included articles are listed in Table 1, while the excluded articles are detailed in Table 2.

The total number of subjects analyzed was 71, as some studies reported multiple ABC cases. These were treated as individual cases in the statistical analysis.

### 2.5. Analyzed Data

The collected data were categorized into three main groups:

- Patient demographics, including gender, age, presence of known genetic disorders, and history of previous head trauma;
- ABC characteristics, including lesion location, size (measured in millimeters), growth trend, symptoms and their relation to the lesion, and etiopathogenesis;
- Management characteristics, including type of treatment, type of surgery, use of preoperative embolization, short- and long-term complications, duration of follow-up (in months), and presence of recurrence.

History of head trauma was defined as any injury to the calvaria that occurred before the onset of the ABC. Cases in which head trauma led to the diagnosis due to lesion rupture and subsequent hemorrhage were not considered as having a trauma-related etiological factor.

As previously mentioned, only ABCs located in neurocranial bones were included, specifically the frontal, parietal, temporal, occipital (including condyles), sphenoid (greater and lesser wings, sella turcica), and ethmoid (cribriform plate) bones.

Growth trend was assessed based on clinical history, particularly any reported enlargement of the lesion during outpatient follow-up.

In terms of etiopathogenesis, we focused on distinguishing between primary ABCs and secondary ABCs, the latter arising from pre-existing fibrous dysplasia (FD) or other congenital/malformative lesions.

Treatment was classified as either conservative management or surgery, with or without adjunctive pharmacotherapy. Surgical procedures were categorized as Gross Total Resection (GTR), Subtotal Resection (STR), or biopsy. In cases where a preoperative biopsy was followed by a GTR, only the GTR was considered for final analysis.

Short-term complications were defined as any adverse events occurring within the first month post-surgery. Complications occurring beyond this period were classified as long-term.

### 2.6. Statistical Analysis

The normality of data distribution was assessed using the Shapiro–Wilk test, which revealed significant deviations from normality (*p* < 0.05) across all datasets. As a result, non-parametric tests were employed for all subsequent analyses. Specifically, the Mann–Whitney U test was used for comparing continuous variables. The significance of frequency distributions in contingency tables was evaluated using the chi-square (χ^2^) test. Only statistically relevant results were reported in detail.

## 3. Case Report

Our patient was a 14-year-old male who presented with acute pain and swelling in his left arm. His medical history included short stature unresponsive to recombinant Growth Hormone (GH) therapy, a traumatic vertebral fracture, and a previous diagnosis of relapsing Acute Lymphoblastic Leukemia (ALL) at the age of 6. He had also recently experienced headaches. Angiographic studies revealed a subclavian artery thrombosis, for which Warfarin therapy was initiated, resulting in clinical improvement.

Physical examination revealed a supernumerary nipple, limb shortening, and bony prominences in the extremities. A full-body X-ray identified multiple exostoses. Genetic testing confirmed a diagnosis of Hereditary Multiple Exostosis (HME) due to an EXT1 gene variant inherited from his mother.

**Figure 2:**
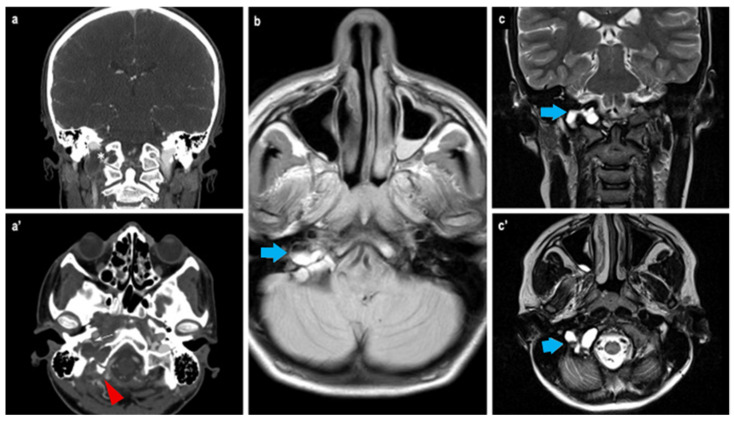
Radiological findings at the CT scan (**a**,**a’**): it is possible to appreciate the bony erosion caused by the extracranial expansive lesion at the right occipital condyle (white asterisk, red arrowhead) with direct contact to the sigmoid sinus. At MRI (**b**,**c**,**c’**), the presence of the characteristic fluid–fluid interface of aneurysmal cyst is clearly visible (blue arrows).

During hospitalization, the patient experienced an acute episode of headache, dizziness, visual disturbances, and a fall. Brain MRI revealed an expansile extra-axial lesion in the right occipital condyle with blood-fluid levels (Figure 2), consistent with a suspected aneurysmal bone cyst (ABC) causing compression of the sigmoid sinus and small ischemic areas in the right cerebellum. Angio-MRI confirmed venous drainage via the left transverse and sigmoid sinuses. Stenosis of the right vertebral artery (VA) and posterior cerebral artery (PCA) was also observed. A cervical spine CT scan revealed small osteochondromas on the cervical vertebrae.

**Figure 3:**
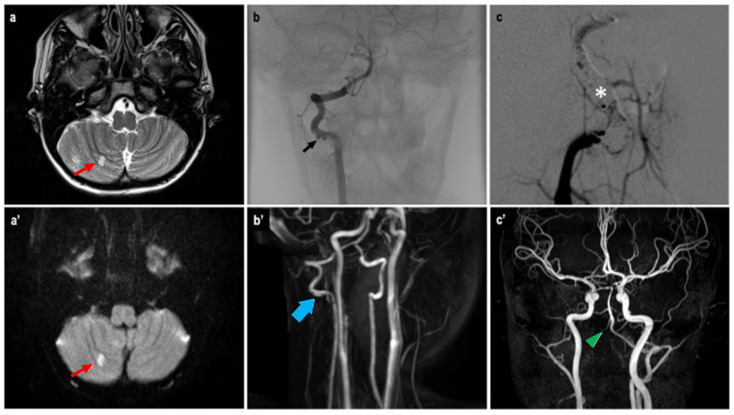
Angio-MRI T2w (**a**) and DWI (**a’**) sequences showing a hyperintense area (red arrow) of the right cerebellar hemisphere, a possible ischemic consequence of the suspected vasospasm of the posterior cerebral circulation. Angiographic study (**b**) confirmed a floating thrombus at the right VA previously described as a VA stenosis at the Angio-MRI (**b’**). A flow-diverter placement was not possible, thus requiring a coiling (white asterisk) of the VA sparing the PICA (**c**). The control Angio-MRI (**c’**) showed the exclusion of the right VA after the endovascular procedure (green arrowhead).

Given the vascular features of the lesion, an angiographic study was conducted. Antiplatelet and anticoagulant therapy were administered preoperatively. During the procedure, reduced blood flow in the left PCA and a floating thrombus in the right VA suggested a vascular dissection (Figure 3). Due to vascular spasm, the placement of a flow diverter stent was not feasible. Instead, the artery was occluded, sparing the right posterior inferior cerebellar artery (PICA), and coils were used to fill the dissected segment. Heparin was administered during the procedure. The neurological outcome was stable postoperatively. Angio-MRI indicated possible vasospasm in the posterior cerebral circulation, prompting continuous heparin infusion.

Due to suspected instability at the CranioCervical Junction (CCJ), a multidisciplinary conference recommended an OccipitoCervical Fixation (OCF) combined with removal of the right occipital condyle. The patient underwent a 4-h surgery with complete en bloc resection of the condyle, which was found to be filled with a dark, hemorrhagic cystic lesion. No major intraoperative bleeding occurred. Fixation included C0-C1-C2, with C1 lateral mass screws, C2 pedicle screws, and an occipital plate.

**Figure 4:**
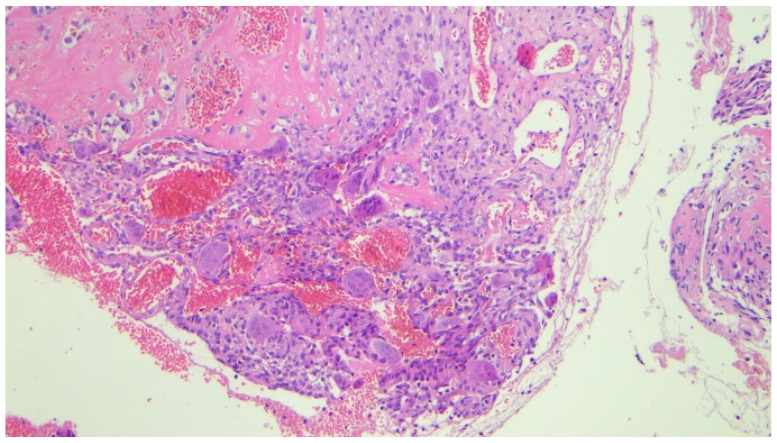
Photomicrograph (hematoxylin-eosin, ×20 magnification) showing numerous multinucleated giant cells filling the wall of the cystic lesion associated with deposition of new bone matrix (upper left).

**Figure 5:**
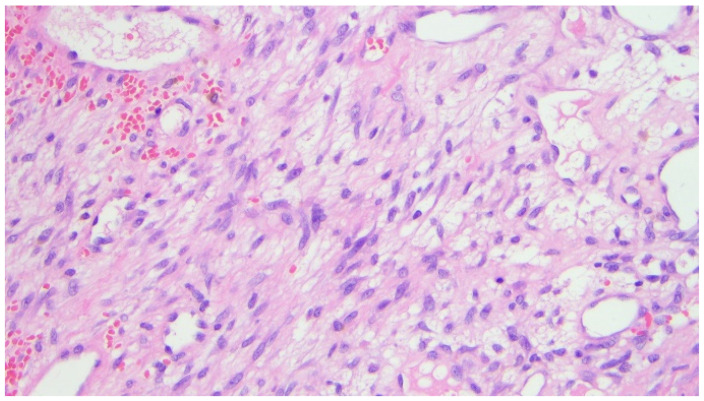
Photomicrograph (hematoxylin-eosin, ×20 magnification) showing associated loose proliferation of spindle cells without significant atypia.

**Figure 6:**
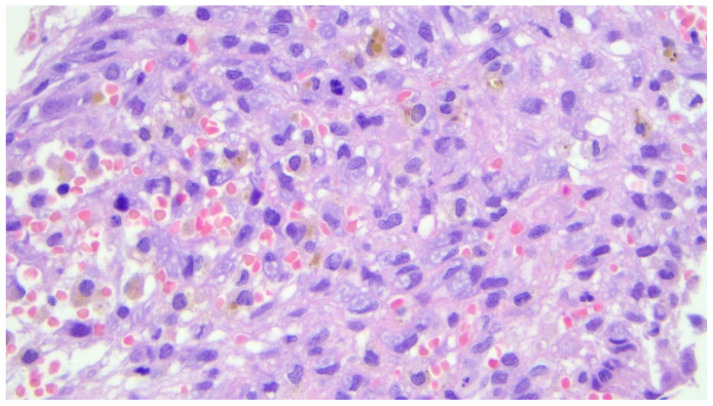
Photomicrograph (hematoxylin and eosin, ×40 magnification) showing the presence of mitosis, which is a common morphologic finding in aneurysmal cysts of the bone.

Histopathological examination revealed numerous multinucleated giant cells lining the cyst wall, new bone matrix deposition (Figure 4), a loosely arranged proliferation of spindle cells without significant atypia (Figure 5), and mitotic figures (Figure 6). The final diagnosis was consistent with an aneurysmal bone cyst.

The postoperative course was uneventful, and the patient was discharged after 14 days in good clinical condition. Radiological follow-up at one year demonstrated optimal fusion of the instrumented segments and no evidence of tumor recurrence. The patient was transitioned to antiplatelet therapy with Aspirin, and Warfarin was resumed.

The patient is currently alive and asymptomatic.

## 4. Results

A total of 74 patients affected by aneurysmal bone cysts (ABCs) were identified in the literature from January 1950 to December 2023, together with our own case report. The mean age at diagnosis was 14.8 ± 12.5 years (range: 2 months to 62 years), with a slightly male gender predominance of 56.8% (Table 3).

The majority of ABCs occurred in the occipital bone (43.2%), followed by the temporal (21.6%), frontal (14.9%), parietal (8.1%), sphenoid (6.8%), and ethmoid (5.4%) bones.

Two cases (2.7%) involved a known genetic disorder: McCune–Albright syndrome [47] and HME (our case report). Thirteen cases (17.6%) showed signs of fibrous dysplasia (FD) of the skull bones on CT scan, of which eight eventually developed into aneurysmal cysts. In two cases (2.7%), the ABC arose from a pre-existing osteoblastoma [59,61], and in one case (1.4%), from a capillary venous malformation [56].

Ten patients (13.5%) reported a history of head trauma prior to the onset of the ABC, while thirty-nine (52.7%) showed a tendency toward progressive growth during follow-up. Fifty-two patients (70.3%) were symptomatic, presenting with compression-related symptoms such as tenderness, swelling, pain, or neurological deficits.

Surgical treatment was performed in 70 cases (94.6%), of which 67 underwent Gross Total Resection (GTR). Among the remaining patients,

- Two were managed conservatively: one refused surgery and was treated with bisphosphonate therapy (alendronate monosodium trihydrate) [34]; the other had surgery postponed due to the COVID-19 pandemic and experienced spontaneous lesion regression [57].
- One underwent an open biopsy, with residual pathological tissue left due to adherence to the clivus and cerebellar structures. This case was managed with corticosteroids and a hormonal blocker [33].
- One underwent a Subtotal Resection (STR) followed by radiation therapy and interferon alfa-2a treatment [37].

Seven patients (9.5%) received preoperative embolization.

Regarding complications, nine patients (12.2%) experienced early postoperative issues, including

- Wound infection [41];
- Headaches and nuchal pressure [33];
- Early recurrence [29];
- Cerebrospinal fluid leakage [54];
- Conductive hearing loss and facial nerve deficit [56];
- Visual deterioration [16];
- Hemifacial numbness [62];
- Cerebral sinus thrombosis [6];
- Cardiac arrest with fatal outcome [7].

No late complications were reported. The mean follow-up period was 24.7 ± 23.2 months, with five recurrences reported at 1 week [29], 2.5 months [22], 4 months [53], and 12 months [52,59], respectively (mean recurrence interval: 0.6 ± 2.5 months).

Statistical analysis was performed, and the statistically significant results are reported in the following paragraphs.

### 4.1. Parietal and Occipital Bone Localization

A statistically significant higher growth trend of aneurysmal bone cysts was observed in lesions located in the parietal bone (*p* = 0.025, Table 4). In contrast, lesions located in the occipital bone were found to have a significantly higher incidence of symptoms and a higher likelihood of developing from fibrous dysplasia in older patients, with *p*-values of 0.007 and 0.019, respectively. Both of these findings were observed exclusively in the male population.

Conversely, no statistically significant correlation was found between fibrous dysplasia and the onset of ABCs (*p* = 0.07), nor between a history of trauma and the development of an ABC (*p* > 0.05).

### 4.2. Early Complications and Etiopathogenesis

**Figure 7:**
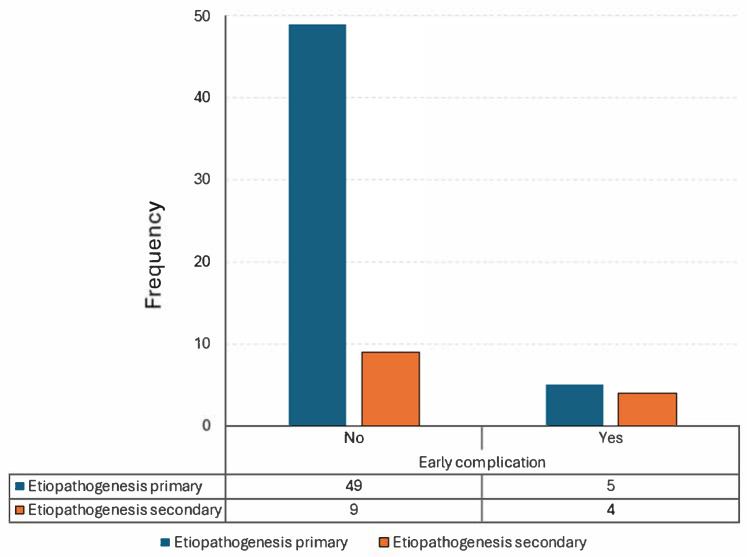
Visual representation of Table 5.

A statistically significant difference in the frequency of early complications was found between patients with primary ABCs and those with secondary lesions (regardless of whether they originated from fibrous dysplasia or other genetic syndromes) (*p* = 0.041, Table 5, Figure 7). This finding indicates that secondary lesions are more likely to develop early complications within the first month after surgery, regardless of their origin.

## 5. Discussion

Originally described by Jaffe and Lichtenstein in 1942 as “solitary unicameral bone cysts” [118], these lesions were formally named aneurysmal bone cysts (ABCs) in 1944 [119]. ABCs are rare, benign, vascular bone tumors, most frequently diagnosed within the first two decades of life [1].

They are histologically complex lesions, composed of blood-filled cystic spaces separated by fibrous stroma containing inflammatory cells, numerous capillaries, and multinucleated giant cells/osteoclasts [2].

The typical location for an ABC is the metaphysis of long bones, although approximately 20% occur in the spine [120]. They account for 1–5% of all primary pediatric bone tumors [121], with a slightly higher incidence in females, as reported in the literature. ABCs involving the calvaria are extremely rare, representing only 3–6% of all ABCs [3].

All cranial bones may potentially be involved, with the jaw—particularly the mandible—being the most commonly affected according to the available literature [122]. However, our study focused exclusively on the neurocranium, where we found the occipital bone (including condylar lesions) to be the most frequently involved site. Notably, patients with occipital lesions tended to become symptomatic at a later age compared to other skull locations—a finding that may suggest slower growth rates, though this was not statistically demonstrated. Moreover, patients with fibrous dysplasia (FD) in the occipital bone were more likely to develop ABCs later in life than those without FD. This may be related to the distinct genetic pathway of FD, which is caused by somatic activating mutations in the GNAS gene, located on chromosome 20q13.3 [122]. FD leads to the replacement of normal bone with fibro-osseous tissue, typically lacking hematopoietic marrow [123], and additional genetic alterations may be required for the development of a vascular lesion such as an ABC, thus necessitating more time for the transformation.

The parietal bone showed a statistically significant higher frequency of growing ABCs, which could be explained by the anatomical and functional context. Lesions in this area may cause early symptoms due to involvement of the temporalis muscle, making them easier to detect. The earlier the diagnosis, the higher the chance of observing lesion growth during follow-up.

Interestingly, both of the above findings—occipital and parietal bone associations—were observed exclusively in the male population. However, no definitive gender predisposition has been confirmed in the literature [124], and to date, no molecular or genetic studies have explored a potential role of male hormones. Future research into the influence of sex hormones on ABC pathophysiology is therefore warranted.

Despite being benign, ABCs can lead to significant bone destruction and mass effect due to their expansile nature, producing symptoms such as pain, swelling, deformity, pathological fractures, and neurological deficits.

Imaging modalities used to assess ABCs include CT scans, which typically show lytic lesions with a honeycomb or eggshell ballooning appearance, and MRI, which reveals characteristic fluid-fluid levels on T2-weighted sequences and a heterogeneous appearance on T1-weighted images [125].

Surgical curettage remains the most common treatment, though recurrence is frequent, often requiring repeat interventions. In cases where lesions are incompletely resectable or inoperable, selective arterial embolization may be the first-line treatment. Other minimally invasive options include radiofrequency ablation, percutaneous injection of demineralized bone matrix, and autologous bone marrow concentrate grafting [126].

In the presented case, embolization was not feasible due to vertebral artery dissection, though the intentional closure of the artery may have contributed to the bloodless surgical field during resection of the right occipital condyle. The rationale for condyle removal was based on CranioCervical Junction (CCJ) instability, with potential for lesion rupture and associated complications.

Despite the availability of numerous surgical options, the clinical course of aneurysmal bone cysts (ABCs) remains unpredictable in some cases, and local recurrences may occur. Recurrence rates have been reported to be as high as 60% in long bones [127], approximately 12.8% in the spine [128], and vary widely in the skull, with estimates ranging from 10% to 50% [129]. In our case series, 5 out of 73 patients (6.9%) experienced a recurrence of the ABC lesion during the follow-up period (the cohort was reduced to 73 from 74 due to one intraoperative death). However, the considerable variability in follow-up duration limited the statistical significance of this finding.

Regarding complication rates, these are generally considered low for curettage alone [130]. The most common complication in long bones is recurrence itself, while long-term complications may include chronic pain, limb-length discrepancies, infection, and heterotopic ossification, although these are considered rare events [131]. At the spinal level, the most common complications are persistent neurological deficits (6.6%), spinal deformity (5.5%), and ongoing pain (2.6%), with an overall low mortality rate of 1.5% [128]. For skull bones specifically, limited data are available in the literature regarding the incidence of complications. In our series, we observed a significant association between early postoperative complications and secondary ABCs, likely reflecting a more complex clinical scenario with increased morbidity and mortality risks.

The etiology of ABCs remains uncertain, with several competing hypotheses. Primary ABCs are widely thought to be reactive lesions, though recent cytogenetic analyses suggest they may be neoplastic. Trauma has been proposed as a possible trigger, but major reviews by Tillman et al. [132] and Ruiter et al. [133] found no supporting evidence—a conclusion echoed by our data (*p* > 0.05).

Another proposed mechanism involves altered osseous circulation, which could occur in patients with increased thrombotic tendency, such as those with Acute Lymphoblastic Leukemia (ALL). Abuzayed et al. discussed this theory in a 2010 case report [40], though the low incidence of ABCs in ALL patients argues against it.

Secondary ABCs, on the other hand, may arise from preexisting lesions such as FD, a rare, benign but chronically progressive bone disorder characterized by the replacement of normal bone with fibrous tissue [134]. FD may be monostotic or polyostotic, and in some cases is associated with endocrinopathies and café-au-lait spots, as seen in McCune-Albright syndrome.

In recent years, molecular studies have revealed a complex genetic landscape underlying aneurysmal bone cysts (ABCs). The most common alteration is a translocation involving the *USP6* gene (also known as *TRE17*) on chromosome 17p13, most frequently with *CDH11* as the fusion partner [135,136,137]. This translocation leads to *USP6* overexpression, which drives tumorigenesis and local bone destruction. As previously mentioned, fibrous dysplasia is associated with mutations in the *GNAS* gene, which encodes the alpha subunit of the stimulatory G protein. A possible hypothesis linking *GNAS* mutations to *USP6* translocations may involve an increased replication rate in affected cells, predisposing them to duplication errors such as gene translocations. However, this hypothesis remains purely speculative, as no supporting studies currently exist.

Other translocations, such as MYH9-USP6, have also been identified [138]. Additionally, somatic mutations in genes related to angiogenesis (e.g., IDH1, IDH2) and osteoclast activity (e.g., PLCG2) have been observed, shedding light on the vascular and osteolytic characteristics of ABCs [139,140].

Our 14-year-old patient was affected by Hereditary Multiple Exostoses (HME), an autosomal dominant disorder marked by multiple osteochondromas. While not observed in our case, the literature reports cases of chondrosarcoma arising in HME patients, sometimes associated with ABCs in long bones. Whether altered bone circulation, osteolytic activity of chondrosarcoma, or both contribute to ABC formation remains unclear. However, ABCs may coexist with a variety of underlying lesions, suggesting the existence of a yet unidentified common molecular or cellular pathway.

## 6. Conclusions

In conclusion, we demonstrated that no significant correlation exists between fibrous dysplasia (FD) and aneurysmal bone cyst (ABC) formation, nor between head trauma and the development of ABCs. However, male patients with FD show a slightly higher tendency to develop ABCs at an older age, specifically in the occipital bone, compared to healthy individuals. Additionally, these patients exhibit a higher tendency for ABCs to grow in the parietal bone. To date, and to the best of our knowledge, no molecular or genetic correlation with male hormones has been reported in the literature. Surgery remains the primary treatment for ABCs, although complications should be carefully considered, especially in the presence of pre-existing pathological conditions.

## 7. Limitations

Our study has several limitations that must be acknowledged. First, aneurysmal bone cysts are rare lesions, and their occurrence in the skull is even less common. This rarity inherently limits the size and representativeness of available case series, including our own, which compromises the statistical power and generalizability of the findings. Second, the majority of published literature on skull ABCs consists of isolated case reports or small case series, often lacking standardized methodology, consistent outcome measures, or long-term follow-up. As such, our ability to draw robust comparisons or establish evidence-based conclusions is restricted.

The retrospective nature of our data collection introduces inherent limitations, including information and selection biases. Medical records, imaging studies, and surgical reports vary in completeness and quality, and the lack of prospective protocols may have led to underreporting or inconsistent documentation of relevant variables such as lesion size, surgical margins, and long-term outcomes.

Moreover, the small number of patients in our series not only limits statistical significance for several variables but also impairs our capacity to perform multivariate analysis to control for potential confounding factors. Some findings, while suggestive, should be interpreted with caution due to the limited sample size.

Another major limitation is the current lack of understanding of the genetic and molecular mechanisms underlying ABC development, especially in secondary forms or in association with other bone pathologies such as fibrous dysplasia. Although we proposed a speculative hypothesis regarding a possible link between *GNAS* mutations and *USP6* translocations, as well as a possible interaction with male hormones, this remains unvalidated and requires experimental support. The absence of molecular data in many of the cases further impedes a mechanistic interpretation of the disease process.

Finally, heterogeneity in diagnostic criteria, treatment approaches, and follow-up duration across the included cases further complicates efforts to identify consistent patterns or risk factors. Differences in imaging modalities, surgical expertise, and institutional protocols may have influenced outcomes in ways that are difficult to quantify retrospectively.

In conclusion, although our study represents one of the more comprehensive reviews of skull ABCs to date, its findings should be considered exploratory. To validate our observations and clarify the biological behavior of these lesions, future prospective studies with larger, multicentric patient cohorts, standardized clinical protocols, and integrated genetic analyses are essential.

## Figures and Tables

**Table 1:** All included articles. * MC: multi-country. N/A: not available. FD: fibrous dysplasia.

Author(s)	Year	Country	N° Patient	Age	Gender	Affected Bone	Symptoms and Signs	Trauma	FD	Treatment	Follow-Up
*Bhende* [5]	1950	India	1	9	M	Temporal bone	Painless nodule	Yes	No	Resection	-
*Blundell* [6]	1965	Australia	1	53	M	Parietal bone	Paralysis of left leg weaking of left arm	No	No	Resection	Deceased
*Odeku* [7]	1965	Nigeria	1	6	M	Occipital bone	Fever Severe occipital headaches Transient vomit	No	No	Resection	Deceased
*Costantini* [8]	1966	Italy	1	14	F	Frontal bone	Vomit Continuous bilateral frontal headaches Bilateral sixth cranial nerve paresis Slight right hemiparesis Papilledema	No	No	Resection	5 yrs
*Cacdac* [9]	1972	USA	1	17	F	Parietal bone	Occasional headaches	No	No	Resection	N/A
*O’Gorman* [10]	1976	Canada	1	1	M	Ethmoid bone	Left proptosis Restriction of eyes movement	No	No	Resection	2 yrs
*Rao* [11]	1977	India	1	14	M	Temporal bone	Headache and neck pain	No	No	Biopsy, resection	N/A
*Mufti* [12]	1978	Iraq	1	25	F	Frontal bone	Symptomless	Yes	No	Resection	N/A
*Luccarelli* [13]	1980	Italy	1	19	F	Occipital bone	Pulsating headache and vertigo Right sixth nerve palsy Ataxic gait	No	No	Resection	1 yr
*Keuskamp* [14]	1980	USA	1	62	M	Temporal bone	Cheyne-Stokes breathing Decerebrate posture Left oculomotor palsy	No	No	Resection	2 mos
*Komjátszegi* [15]	1981	Romania	1	11	M	Occipital bone	Symptomless	Yes	No	Resection	5 yrs
*Kimmelman* [16]	1982	USA	1	10	M	Sphenoid bone	Decreased vision	No	No	Two stages resection	4 mos
*Baker* [17]	1982	USA	1	20	F	Ethmoid bone	Nasal obstruction Periorbital swelling with mild proptosis Blurred vision and occasional diplopia	No	No	Biopsy, resection	2 yrs
*Sanerkin* [18]	1983	UK	1	5	M	Ethmoid bone	Proptosis right eye	No	No	Resection	3 yrs
*Bilge* [19]	1983	Turkey	2	3, 18	F, M	Occipital bone	Headaches, vomit and vertigo Papilledema Bilateral sixth nerve palsies, cerebellar signs	No	No	Resection	1.5 yrs, 1 yr
*Ameli* [20]	1984	Albania	4	13, 16, 25, 20	F, F, M, M	Frontal, sphenoid, frontal, parietal bone	Headache Exophthalmos	Yes, no, no, yes	No	Resection	5 yrs, 6 yrs, N/A, 1 yr
*Calliauw* [21]	1985	Belgium	3	2 mos, 36, 19	M, M, F	Parietal, temporal, temporal bone	Deafness Facial palsy and sensory loss Pain	No	No	Resection	2 yrs, 2.5 yrs, 1.5 yrs
*Braun* [22]	1987	Israel	1	4	F	Occipital bone	Symptomless	No	No	Resection, radiotherapy	4 mos
*Arthur* [23]	1988	France	2	9, 9	F, M	Occipital, frontal bone	Ophthalmoplegia, exophthalmos, papilledema	Yes, no	No	Resection, embolization and resection	N/A
*Rappaport* [24]	1989	Israel	1	25	M	Occipital bone	Pain	Yes	Yes	Resection	N/A
*Hunter* [25]	1990	UK	1	7	M	Sphenoid bone	Periorbital swelling and proptosis	No	No	Biopsy, resection	1 yr
*Dávid* [26]	1993	Hungary	1	21	M	Occipital bone	Headaches, nausea, dizziness, and vomiting	No	No	Resection	3 yrs
*Lau* [27]	1995	USA	1	15	M	Occipital bone	Nausea and visual changes	No	No	Resection	N/A
*Chateil* [28]	1997	France	2	7, 9	M, F	Temporal, occipital bone	Diplopia, headache, vomit, sixth nerve palsy	No, yes	No	Resection, biopsy and resection	N/A
*Park* [29]	1999	Canada	1	4	M	Temporal bone	Otorrhea with otalgia	No	Yes	Resection	1 yr
*Petro* [30]	2001	USA	1	7	F	Occipital bone	Headache and hydrocephalus	No	No	Resection	N/A
*Roncaroli* [31]	2001	Italy	1	2	M	Occipital bone	Symptomless	No	No	Resection	N/A
*Itshayek* [32]	2002	Israel	1	19	M	Occipital bone	Symptomless	No	Yes	Biopsy, embolization, resection	1 yr
*Mattei* [33]	2005	Brazil	1	19	F	Parietal and occipital bone	Headache, nuchal rigidity, SAH	No	Yes	Subtotal resection, administration of Zumeta and a hormonal blocker	N/A
*Iseri* [34]	2005	Turkey	1	35	F	Occipital bone	Gait disturbance, swallowing difficulty, slurring of speech, vertigo, altered sensation of hands	No	Yes	Administration of Alendronate	N/A
*Lin* [35]	2007	Taiwan	1	54	F	Occipital bone	Symptomless	No	Yes	Resection	5 mos
*Gan* [3]	2007	UK	3	5, 8, 4	F, M, F	Temporal, occipital, temporal bone	Exophthalmos, double vision	No	No	Biopsy and resection, resection, biopsy and resection	3 yrs, 3 yrs, 4 yrs
*Han* [36]	2008	China	1	20	M	Occipital	Headache	No	No	Resection	18 mos
*Segall* [37]	2008	Canada	4	4, 7, 7, 5 mos	M, M, F, F	Temporal, parietal, frontal, ethmoid	Pain, facial paresis, visual loss with proptosis, orbital deformity	No	No	Resection, resection, embolization and resection, resection	10 yrs, 5 yrs, 3 yrs, 2.5 yrs
*Gopalakrishnan* [38]	2009	India	1	14	M	Sphenoid bone	Headache, focal seizure	No	No	Resection	N/A
*Lee* [39]	2010	South Korea	1	18	F	Frontal bone	Headache	No	Yes	Resection	N/A
*Abuzayed* [40]	2010	Turkey	1	11	F	Frontal bone	Symptomless	No	No	Resection	N/A
*Krishnan* [41]	2011	USA	1	8	F	Occipital bone	Symptomless	No	No	Resection	6 mos
*Genizi* [42]	2011	Israel	1	2	M	Occipital bone	Headache and stumbling	No	No	Resection	6 mos
*Umredkar* [43]	2012	India	1	8	M	Occipital bone	Pain	No	No	Resection	6 mos
*Curtis* [44]	2012	USA	1	16	M	Occipital bone	Obstructive hydrocephalus	No	No	Resection	N/A
*Garber* [45]	2015	USA	1	3	F	Occipital bone	Vomited, lethargic, GCS 10	Yes	No	Resection	6 mos
*Kalina* [46]	2015	USA	1	9	M	Occipital bone	Headache, emesis, dizziness	No	No	Resection	N/A
*Urgun* [47]	2016	USA	1	14	F	Occipital bone	Symptomless	No	Yes	Resection	N/A
*Spina* [48]	2016	Italy	1	22	N/A	Occipital condyle	Neck pain	No	No	Resection	3 yrs
*Mete* [49]	2017	Turkey	1	12	F	Occipital bone	Headache and vomit	Yes	No	Resection	N/A
*Chaurasia* [50]	2018	Bangladesh	1	19	F	Occipital bone	Headache	No	No	Resection	N/A
*Alves Filho* [51]	2019	Brazil	1	17	F	Sphenoid bone	Headache	No	No	Biopsy	N/A
*Brohi* [52]	2019	Pakistan	1	50	M	Temporal bone	Headache, facial nerve palsy	No	No	Resection	1 yr
*Tse* [53]	2020	MC *	1	11	F	Occipital bone	Headache, nystagmus	Yes	No	Resection, embolization, gamma knife	8 yrs
*Gotecha* [54]	2020	India	1	3	M	Temporal bone	Symptomless	No	No	Resection	N/A
*Farouk* [55]	2020	Nigeria	1	7	M	Occipitoparietal bone	Headache	No	No	Resection	N/A
*Canzano* [56]	2021	Italy	1	5	M	Temporal bone	Facial palsy	No	No	Embolization, resection	2 yrs
*Borni* [57]	2022	Tunisia	1	7	M	Frontal bone	Symptomless	No	No	Spontaneous regression	3 mos
*Woldow* [58]	2022	USA	1	14	M	Temporal bone	Symptomless	No	No	Resection	N/A
*An* [59]	2023	China	1	16	F	Temporal bone	Headache and dizziness	No	No	Resection	1 yr
*Koketsu* [60]	2023	Japan	1	19	F	Frontal bone	Symptomless	No	No	Resection	N/A
*Alsabbagh* [61]	2023	KSA	1	9	M	Occipital bone	Headache, nausea, vomit, hydrocephalus	No	No	EVD placement and resection	N/A
*Shen* [62]	2023	Taiwan	1	34	M	Temporal bone	Otorrhea, mild tinnitus, hearing loss, and occasional headache	No	No	Resection	N/A
*Mousavinejad* [63]	2023	Iran	1	17	M	Frontal bone	Symptomless	No	No	Resection	2 yrs
*Our case report*	2025	Italy	1	14	M	Occipital condyle	Headache, dizziness, impaired vision, and fall	No	No	Resection	1 yr

**Table 2:** Excluded reports.

Author(s)	Year	Country	Reason for Exclusion
*Oki* [64]	1978	Japan	Not-English article
*Mima* [65]	1984	Japan	Not-English article
*Ikeda* [66]	1985	Japan	Not-English article
*Tokarz* [67]	1993	Poland	Not-English article
*Arnaldsson* [68]	1995	Iceland	Not-English article
*Clavel* [69]	2001	Spain	Not-English article
*Nakaoka* [70]	2002	Japan	Not-English article
*Mazlout* [71]	2005	France	Not-English article
*Broc-Haro* [72]	2007	Spain	Not-English article
*Wendt* [73]	2010	Germany	Not-English article
*Abdoulkader* [74]	2017	France	Not-English article
*Zhang* [75]	2021	China	Not-English article
*Ao* [76]	2022	China	Not-English article
*Bull* [77]	2024	USA	Animal study
*Syed* [78]	2025	USA	No case report reported
*Hino* [79]	1998	Japan	Radiological study
*Yarington* [80]	1964	USA	Anatomical location outside the skull
*Fite* [81]	1968	USA	Anatomical location outside the skull
*O’Gorman* [10]	1976	Canada	Anatomical location outside the skull
*Eveson* [82]	1978	UK	Anatomical location outside the skull
*Rasi* [83]	1978	USA	Anatomical location outside the skull
*Matt* [84]	1993	USA	Anatomical location outside the skull
*Winters* [85]	1998	Belgium	Anatomical location outside the skull
*Senol* [86]	2002	Turkey	Anatomical location outside the skull
*Sánchez* [87]	2004	Mexico	Anatomical location outside the skull
*Perrotti* [88]	2004	Italy	Anatomical location outside the skull
*Smolka* [89]	2008	Switzerland	Anatomical location outside the skull
*Pelo* [90]	2009	Italy	Anatomical location outside the skull
*Roychoudhury* [91]	2009	India	Anatomical location outside the skull
*Bozbuğa* [92]	2009	Turkey	Anatomical location outside the skull
*Sun* [93]	2009	China	Anatomical location outside the skull
*Breuer* [94]	2010	Germany	Anatomical location outside the skull
*Choi* [95]	2011	Republic of Korea	Anatomical location outside the skull
*Lee* [96]	2012	Republic of Korea	Anatomical location outside the skull
*Verma* [97]	2013	India	Anatomical location outside the skull
*Simsek* [98]	2013	Turkey	Anatomical location outside the skull
*Lerant* [99]	2013	Hungary	Anatomical location outside the skull
*Janjua* [100]	2014	UK	Anatomical location outside the skull
*Lee* [101]	2014	Republic of Korea	Anatomical location outside the skull
*Neuschl* [102]	2014	Germany	Anatomical location outside the skull
*Bhandari* [103]	2015	India	Anatomical location outside the skull
*Wang* [104]	2015	China	Anatomical location outside the skull
*Liu* [105]	2017	China	Anatomical location outside the skull
*Toescu* [106]	2017	UK	Anatomical location outside the skull
*Arocho* [107]	2018	USA	Anatomical location outside the skull
*Asi* [108]	2018	USA	Anatomical location outside the skull
*Bajpai* [109]	2018	India	Anatomical location outside the skull
*Rațiu* [110]	2018	Romania	Anatomical location outside the skull
*Smith* [111]	2021	USA	Anatomical location outside the skull
*Foo* [112]	2022	USA	Anatomical location outside the skull
*Yahaya* [113]	2023	Tanzania	Anatomical location outside the skull
*Mo* [114]	2023	USA	Anatomical location outside the skull
*Phan* [115]	2023	Australia	Anatomical location outside the skull
*O’Leary* [116]	2024	USA	Anatomical location outside the skull
*Oh* [117]	2024	Republic of Korea	Anatomical location outside the skull

**Table 3:** Summary of the total collected data. The mean age is in years. Mean follow-up is in months. No gender predominance was seen. Genetic disorder was considered any condition genetically diagnosed. Fibrous dysplasia was considered only in the skull bone. Growth tendency refers to the increase in the lesion’s volume reported by the patient or documented by imaging. Any symptoms related to the cyst were considered, like tenderness, swelling, pain, or neurological deficit. Surgery was divided into Gross Total Resection (GTR), Subtotal Resection (STR), and biopsy. * Lesion size is calculated as the major diameter in millimeters.

N° Patients	Mean Age	Male(s)	Genetic Disorders	Fibrous Dysplasia	Previous Trauma	Growth Tendency
74	14.8 ± 12.5 yrs	42	2	13	10	39
Symptomatic	Mean follow-up	GTR	STR	Biopsy	Conservative	Embolization
52	24.7 ± 23.2 mos	67	7	0	2	7
N° early complications	Early complications		N° late complications	Recurrence	Time of recurrency	* Lesion size
9	Occipital bone: Cardiac arrest with exitus; Headache and nucal pressure. Wound infection Temporal bone: CSF otorrhea; Earing loss and facial palsy; Hemifacial numbness; Early recurrency. Parietal bone: Cerebral sinus thrombosis. Sphenoid bone: Visual deterioration.		0	5	0.6 ± 2.5 mos	64 mm
Occipital bone	Temporal bone	Frontal bone	Parietal bone	Ethmoidal bone	Sphenoid bone	
32	16	11	6	4	5	

**Table 4:** Contingency table showing a statistically significant higher frequency in growth trend of aneurysmal cysts at the parietal bone in the male population alone.

Contingency Tables				
		Growth Trend:		
Location:	Gender:	No	Yes	Total
occipital	female	10	6	16
	male	7	9	16
	Total	17	15	32
temporal	female	1	3	4
	male	5	7	12
	Total	6	10	16
frontal	female	3	4	7
	male	3	1	4
	Total	6	5	11
parietal	female	1	0	1
	male	1	4	5
	Total	2	4	6
ethmoidal	female	1	1	2
	male	0	2	2
	Total	1	3	4
sphenoid	female	1	1	2
	male	2	1	3
	Total	3	2	5
Total	female	17	15	32
	male	18	24	42
	Total	35	39	74
*Chi-Squared Tests*				
Location:	Value		df	*p*
occipital	Χ^2^	1.200	1	0.273
	N	30		
temporal	Χ^2^	0.280	1	0.597
	N	14		
frontal	Χ^2^	1.102	1	0.294
	N	9		
parietal	Χ^2^	5.000	1	0.025
	N	5		
ethmoidal	Χ^2^	NaN		
	N	3		
sphenoid	Χ^2^	0.000	1	1.000
	N	4		
Total	Χ^2^	0.961	1	0.327
	N	65		

**Table 5:** Contingency table showing a statistically significant difference in frequency of the onset of early complications between patients with a primitive ABC and those with a secondary lesion.

Contingency Tables.			
	Early Complications:		
Etiopathogenesis	No	Yes	Total
primary	49	5	54
secondary	9	4	13
Total	58	9	67
*Chi-Squared Tests*			
	Value	df	*p*
Χ^2^	4.169	1	0.041
N	67		

Note: each cell displays the observed counts.

## Data Availability

The data that support the findings of this study are available from the corresponding author, Leonardo Bradaschia, upon reasonable request.

## Abbreviations

The following abbreviations are used in this manuscript:

PRISMA	Preferred Reporting Items for Systematic Reviews and Meta-Analyses
ABC	aneurysmal bone cysts
FD	fibrous dysplasia
GH	Growth Hormone
ALL	Acute Lymphoblastic Leukemia
HME	Hereditary Multiple Exostosis
PCA	Posterior Cerebral Artery
PICA	Posterior Inferior Cerebellar Artery
CCJ	CranioCervical Junction
OCF	OccipitoCervical Fixation
GTR	Gross Total Resection
STR	Sub Total Resection
MRI	Magnetic Resonance Imagining
MC	multi-country
CT	computed tomography
DWI	Diffusion-Weighted Imaging
